# A new species of *Amphoropsyche* (Trichoptera, Leptoceridae) from Ecuador, with a key to the species in the genus

**DOI:** 10.3897/zookeys.111.813

**Published:** 2011-06-22

**Authors:** Ralph W. Holzenthal, Luis Ernesto Rázuri-Gonzales

**Affiliations:** 1 University of Minnesota, Department of Entomology, 1980 Folwell Ave., 219 Hodson Hall, St. Paul, Minnesota 55108, U.S.A.; 2 Departamento de Entomología, Museo de Historia Natural, Universidad Nacional Mayor de San Marcos, Av. Arenales 1256, Apartado Postal 14–0434, Lima, Perú

**Keywords:** Trichoptera, Leptoceridae, *Amphoropsyche*, caddisfly, new species, Neotropics, Ecuador, key to species

## Abstract

A new species of *Amphoropsyche* Holzenthal is described from Ecuador. It is similar to a group of species with dorsomesal processes on the preanal appendages (i.e., *Amphoropsyche woodruffi* Flint & Sykora, *Amphoropsyche refugia* Holzenthal, and *Amphoropsyche aragua* Holzenthal), but can be distinguished from these and other members of the genus by the short, digitate dorsomesal processes on the preanal appendages and the broad lateral processes of tergum X of the male genitalia. A key to males of the 14 species now known in the genus is presented based on characters of the genitalia.

## Introduction

Flint (1968) described the male, female, and larva of a new species of longhorn caddisfly (Leptoceridae, Leptocerinae) from Dominica, Lesser Antilles, and tentatively placed it in the Chilean genus *Brachysetodes*, based mainly on wing venation. He also noted that the structure of the male genitalia seemed quite different between the Dominican species, *Brachysetodes insularis* Flint, and the type species, *Brachysetodes trifidus* Schmid. He suggested that the discovery of the larva of the Chilean species might show that the 2 species are not congeneric. Based on character differences in the male genitalia, Holzenthal (1985) removed *Brachysetodes insularis* from *Brachysetodes* and established the genus *Amphoropsyche* for Flint’s species along with 9 new species from Ecuador, Colombia and Venezuela. In a separate paper, Holzenthal (1986a) described the immature stages of *Brachysetodes*, confirming Flint’s earlier supposition that *Brachysetodes* and *Amphoropsyche* are distinct. Holzenthal (1986b) also described 1 additional species from Bolivia, extending the known geographical range of the genus considerably southwards, and redescribed the larva of the Dominican species. Since the mid 1980s, only 3 additional nominal species were described, all from the Lesser Antilles and Tobago: *Amphoropsyche janstockiana* Botosaneanu, 1990 from Saint Vincent, *Amphoropsyche multispinosa* Botosaneanu, 1993 (in Botosaneanu and Alkins-Koo 1993) from Trinidad, and *Amphoropsyche woodruffi* Flint & Sykora, 1993 from Grenada (subsequently recorded from northern Venezuela by Flint 1996). Flint (1996) considered *Amphoropsyche multispinosa* to be a geographical variant of *Amphoropsyche woodruffi* and changed its status to a subspecies of the latter. In the same paper, Flint (1996) noted the presence of a single female specimen from Tobago with distinctive genitalia probably representing yet another new species; males have still not been collected and the species remains undescribed.

This genus seems to be especially species-rich in mid-elevation streams (e.g., 1500–2500 m) in the northern Andes and more species are expected to be collected and described (Holzenthal 1986b; Flint et al. 1999). *Amphoropsyche* is characterized by the presence of large glands inside the preanal appendages (probably producing pheromones), and the presence of a tuft of strong hairs near the apex of the inferior appendage, probably involved in dispersing these pheromones (Botosaneanu 1990). In this paper, we describe a new species of *Amphoropsyche*, the 14th in the genus, and provide a key to the males of the species.

## Materials and methods

This study is based on a single pinned specimen collected in Ecuador by Dr. Oliver S. Flint, Jr., National Museum of Natural History, Smithsonian Institution, and kindly loaned to the first author. Techniques and procedures used in the preparation and examination of the specimen are those outlined by Blahnik and Holzenthal (2004) and Blahnik et al. (2007). The illustration of the genitalia was prepared from a pencil sketch made using a camera lucida mounted on an Olympus BX41 compound microscope. The pencil sketch was then scanned and placed into an Adobe Illustrator (version CS5, Adobe Systems, Inc.) document, to serve as a template, and then traced to create a vector graphic illustration. A graphic tablet and pen (BAMBOOTM, Wacom Technology Co.) facilitated careful tracing of the original image.

Terminology used in describing male genitalia follows that of (Holzenthal (1985, 1986b). The taxonomic key was based on published illustrations and descriptions of the male genitalia (Holzenthal 1985, 1986b; Botosaneanu 1990; Flint and Sykora 1993, Botosaneanu and Alkins-Koo 1993; Flint 1996 [these papers can be downloaded from the Trichoptera Literature Database at www.trichoplit.umn.edu to facilitate comparisons]) and was constructed using the DELTA system which facilitated taxonomic data coding via the Delta editor v. 1.04 (Dallwitz 1980; Dallwitz et al. 1999 onwards).

The type is deposited in the United States National Museum of Natural History, Smithsonian Institution, Washington, D.C. (NMHN).

## Species description

### Amphoropsyche tandayapa

Holzenthal & Rázuri-Gonzales sp. n.

urn:lsid:zoobank.org:act:405CE4BA-B14D-4CEB-827E-BFE295FD12F0

http://species-id.net/wiki/Amphoropsyche_tandayapa

Fig. 1A–D 

#### Description.

This species is characterized by the short, digitate dorsomesal processes of the inferior appendages, the long basoventral projection of the 1st article of the inferior appendages, and the broad, lateral processes of segment X. It is most similar to that group of species also possessing dorsomesal processes on the preanal appendages(i.e., *Amphoropsyche woodruffi*, *Amphoropsyche refugia*, *Amphoropsyche aragua*), but differs in having much shorter processes that are unsclerotized.

**Figure 1.**
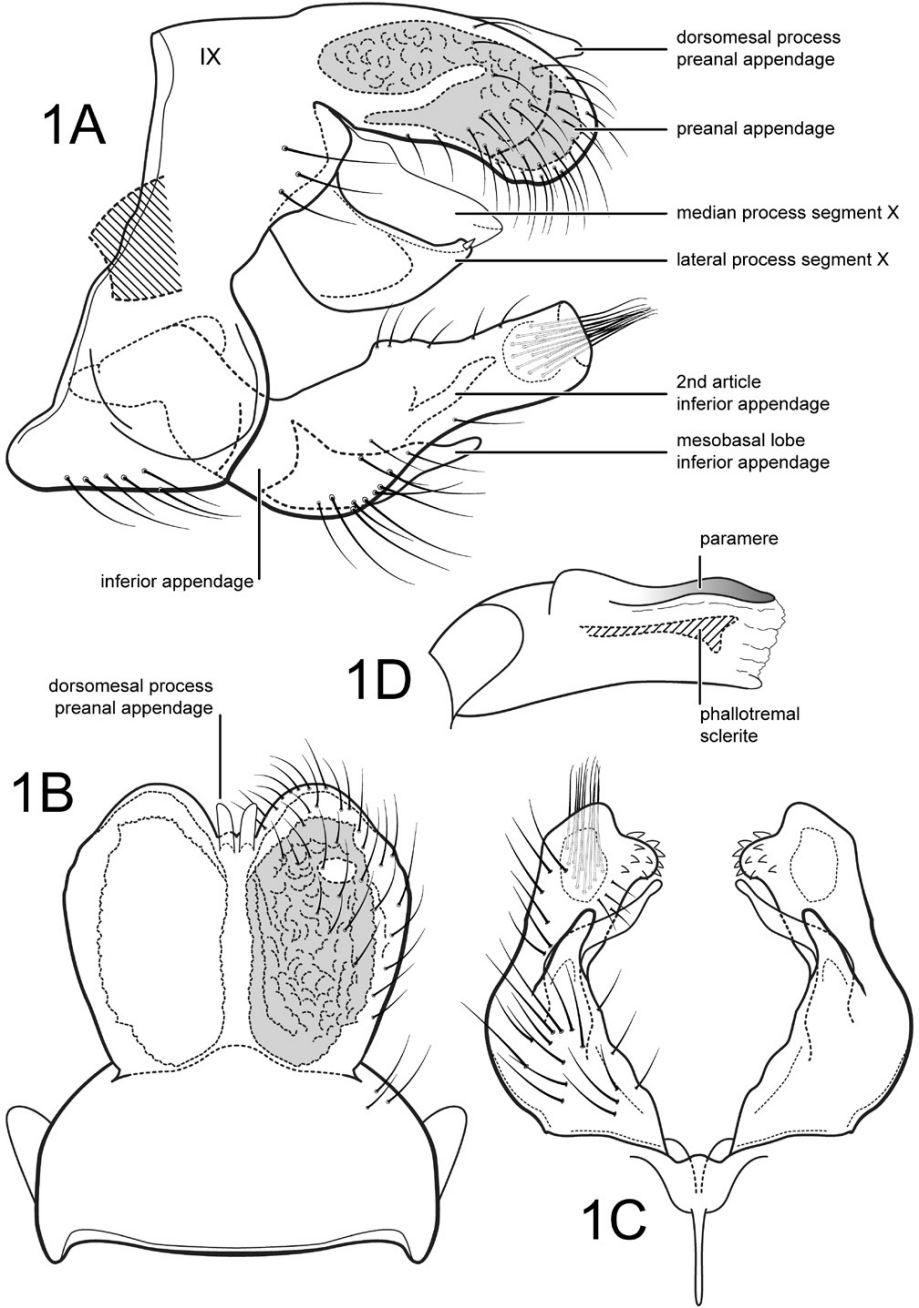
*Amphoropsyche tandayapa*, sp. n. Male genitalia **A** lateral **B** segments IX–X, dorsal **C** inferior appendages, ventral **D** phallus, lateral.

Male. Forewing length 4.8 mm. Wings and body color brown. Genitalia as in Fig. 1 A–D. Segment IX annular, sternum with anterior part slightly extended anteriorly. Segment X composed of a single mesal process and pair of lateral processes; mesal process lightly sclerotized, apex broadly acute; lateral process broad, bearing apical spine-like setae. Preanal appendages large, oval, almost completely fused along their midlengths, with pair of short, digitate, membranous dorsomesal processes; preanal appendage with large reticulate internal gland with small ventral opening. Inferior appendage elongate, with long basoventral projection; inferior appendage angulate basally in lateral view, bent inwards in ventral view, bearing very short spine-like setae on slightly protruding apicomesal corner; 2nd article of inferior appendage elongate, thin, sinuate, slightly curved inwards, apex narrow, rounded. Phallic apparatus with phallobase well developed; pair of dorsal parameres present; phallotremal sclerite well developed, elongate, widest apically in lateral view.

Female and larva: Unknown.

#### Holotype male.

**ECUADOR: Pichincha:** 2.3 km S Tandayapa, 1800 m, 6.x.1990, O.S. Flint, Jr. (NMNH).

#### Etymology.

Named after the town of Tandayapa, located near where the holotype was collected.

## Key to the males of Amphoropsyche

**Table:** 

1	Preanal appendages completely (Holzenthal 1985, Figs 8B, 10B; Flint and Sykora 1993, Fig. 20) or almost completely fused mesally (if the latter, apical emargination shallow, obtuse) (Fig. 1B; Holzenthal 1985, Fig. 3B)	2
–	Preanal appendages not fused mesally, divided to 1/3 to 2/3 of their length (apical emargination acute) (Holzenthal 1985, Figs 5B, 6B)	6
2(1)	Preanal appendages with dorsomesal process (Holzenthal 1985, Figs 8B, 10B)	3
–	Preanal appendages without dorsomesal process (Holzenthal 1985, Figs 3A–D)	*Amphoropsyche insularis*
3(2)	Dorsomesal process of preanal appendages short, digitate, not exceeding length of preanal appendage; dorsomesal processes of preanal appendages not sclerotized (Figs 1 A–D)	*Amphoropsyche tandayapa* sp. n.
–	Dorsomesal process of preanal appendages long, exceeding length of preanal appendage (Holzenthal 1985, Figs 8A, 10A; Flint and Sykora 1993, Fig. 18); dorsomesal processes of preanal appendages sclerotized	4
4(3)	Second article of inferior appendages elongate, narrow (Holzenthal 1985, Fig. 8A)	5
–	Second article of inferior appendages short (Flint and Sykora 1993, Figs 18–20; Botosaneanu and Alkins-Koo 1993, Figs 97–101)	*Amphoropsyche woodruffi*
5(4)	Dorsomesal process of preanal appendages bifid in dorsal view; ventral subterminal portion of phallobase serrate (Holzenthal 1985, Figs 8A–D)	*Amphoropsyche refugia*
–	Dorsomesal process of the preanal appendages entire in dorsal view; ventral subterminal portion of phallobase entire (Holzenthal 1985, Figs 10A–D)	*Amphoropsyche aragua*
6(1)	Second article of inferior appendages present (Holzenthal 1985, Fig. 5A)	7
–	Second article of inferior appendages absent (Holzenthal 1985, Fig. 16C)	13
7(6)	Tergum X with median process and paired lateral processes (Holzenthal 1985, Figs 5A, 14A)	8
–	Tergum X without median process, lateral processes with apical and subapical spinelike projections (Botosaneanu 1990, Figs 1–3)	*Amphoropsyche janstockiana*
8(7)	Second article of inferior appendages short (Holzenthal 1985, Fig. 14C) or long, but broad (Holzenthal 1985, Fig. 6C)	9
–	Second article of inferior appendages elongate and narrow (Holzenthal 1985, Fig. 7C)	11
9(8)	Phallus without parameres (Holzenthal 1985, Fig. 6D)	10
–	Phallus with parameres (Holzenthal 1985, Figs 14A–D)	*Amphoropsyche quebrada*
10(9)	Second article of inferior appendages short, with apical spine-like seta; lateral process of tergum X with subapical spine-like seta; phallicata with pair of bifid, spiniferous, lateral extensions (Holzenthal 1986b, Figs 1A–D)	*Amphoropsyche spinifera*
–	Second article of inferior appendages long, but broad, without apical spine-like seta; lateral process of tergum X with several apical spine-like setae; phallicata without lateral, bifid extensions, but phallobase with ventral spine-like process (Holzenthal 1985, Figs 6A–D)	*Amphoropsyche flinti*
11(8)	Phallus with parameres (Holzenthal 1985, Fig. 5D)	12
–	Phallus without parameres (Holzenthal 1985, Figs 11A–D)	*Amphoropsyche choco*
12(11)	Lateral process of tergum X U-shaped, tip bifid, bearing small spine-like setae (Holzenthal 1985, Figs 5A–D)	*Amphoropsyche napo*
–	Lateral process of tergum X tapered to a sharp terminal point, without spine-like setae (Holzenthal 1985, Figs 7A–D)	*Amphoropsyche stellata*
13(6)	Parameres small; inferior appendage with basoventral lobe (Holzenthal 1985, Figs 16A–D)	*Amphoropsyche cauca*
–	Parameres large; inferior appendage without basoventral lobe (Holzenthal 1985, Figs 12A–D)	*Amphoropsyche ayura*

## Supplementary Material

XML Treatment for Amphoropsyche tandayapa
